# How authenticity impacts validity: Developing a model of teacher education assessment and exploring the effects of the digitisation of assessment methods

**DOI:** 10.1007/s11618-023-01154-y

**Published:** 2023-06-07

**Authors:** Christoph Kulgemeyer, Josef Riese, Christoph Vogelsang, David Buschhüter, Andreas Borowski, Anna Weißbach, Melanie Jordans, Peter Reinhold, Horst Schecker

**Affiliations:** 1 Fakultät für Naturwissenschaften, Department Physik, Universität Paderborn, Warburger Str. 100, 33098 Paderborn, Germany; 2 I. Phys. Institut IA, RWTH Aachen University, Sommerfeldstr. 14, 52074 Aachen, Germany; 3 Institut für Physik und Astronomie, Lehrstuhl Didaktik der Physik, Universität Potsdam, Karl-Liebknecht-Str. 24/25, 14476 Potsdam-Golm, Germany; 4 Institut für Didaktik der Naturwissenschaften, Abt. Physikdidaktik, Universität Bremen, Otto-Hahn-Allee 1, 28334 Bremen, Germany

**Keywords:** Professional competence, Digital assessment, Authenticity, Science education, Professionelle Handlungskompetenz, Digitale Testformate, Authentizität, Physikdidaktik

## Abstract

Based on a literature review of studies on teachers’ professional competence and related assessment tools, this paper introduces a model of teacher education assessment. It is influenced by Miller’s (1990) framework of assessment in medical education and includes, among other aspects, performance assessments. This model is used to understand the potential effects of transferring assessment tools into a digital format with assessment feedback. Five examples for such a transfer will be discussed: three methods for various aspects of communication, a test for pedagogical content knowledge, and a test for content knowledge. All five are established instruments well-described in terms of validity. All five have recently been transferred into a digital format. The analysis of this transfer also reveals a potentially harmful effect of digital assessment. The closer an assessment instrument is to assessing action-related parts of professional competence, the more authenticity is required; however, digitisation tends to decrease this authenticity. This suggests that an increasing number of digital assessment tools in teacher education might result in an even more dominant focus on knowledge tests, ignoring other parts of professional competence. This article highlights the role of authenticity in validity and discusses the most suitable assessment format to address various parts of professional competence. It ends by highlighting the lessons learned from the transfer of assessment instruments into a digital format that other academic disciplines might find interesting.

## Abstract (de)

Im vorliegenden Beitrag soll ein Modell entwickelt werden, das Testformate und zugrundeliegende Fähigkeitsbereiche der professionellen Handlungskompetenz von Lehrkräften zusammenhängend beschreibt. Grundlage dafür ist Millers (1990) analoges Modell für den Bereich der Medizin. Es werden einleitend Studien zur professionellen Kompetenz angehender Physiklehrkräfte analysiert und die dazugehörigen Testinstrumente diskutiert. Kern des Modells ist es dann, die Breite der zu erhebenden Fähigkeitsbereiche zu beschreiben; dabei werden z. B. Performanztests berücksichtigt. Das Modell wird dann dazu verwendet, an fünf Beispielen die Effekte zu beschreiben, die die Digitalisierung von Testformaten auf die erhobenen Aspekte professioneller Handlungskompetenz angehender Lehrkräfte hat. Es zeigt sich ein potenzielles Problem: je handlungsnäher der zu erhebende Aspekt professioneller Handlungskompetenz ist, desto authentischer zum Berufsalltag müssen eigentlich die Problemsituationen werden, die als Kontexte im Test verwendet werden. Die digitalen Formate allerdings führen tendenziell dazu, dass die Authentizität gesenkt wird. Dies könnte darin resultieren, dass mit einer zunehmenden Anzahl digitaler Testformate die bestehende Dominanz des Assessments des Professionswissens erhöht wird und andere Aspekte professioneller Handlungskompetenz in den Hintergrund rücken. Am Ende des Beitrags wird kurz die Rolle von Authentizität für Validität diskutiert und verschiedene „lessons learned“ abgeleitet, die von Interesse für andere akademische Disziplinen der Lehrkräftebildung sein könnten.

## Introduction

Using assessment methods is not only an essential part of research, but also of education. It contributes to various aspects of the educational system, including the evaluation of learning environments and supporting learners’ competence development, for example, by providing assessment feedback (Casanova et al. 2021; Sippel 2009). It also contributes to selecting the most promising students in cases of limited educational resources (Zimmerhofer and Trost 2008). *Digital* assessment methods have been used increasingly over the last decade, most recently accelerated by the COVID-19 pandemic and distance learning. Both for teaching and research purposes, digital assessment is used in general for larger groups of learners, because of its potential to rate answers automatically (e.g., by using AI) and provide immediate automated assessment feedback, thus reducing the time and effort needed for scoring (e.g. Wall 2000).

In teacher education, the area of interest regarding assessment is the broad idea of professional competence. Here, competence is understood as a continuum from professional knowledge to observable performance (as described in Blömeke et al. 2015), with knowledge being a disposition for performance. Some scholars identified a need for methods to assess the performance-related aspects of competence (Aufschnaiter and Blömeke 2010; Grossman et al. 2009; Kulgemeyer and Riese 2018; Schröder et al. 2020). Indeed, most studies focus on assessing knowledge, which might overestimate disposition and underestimate other aspects of competence. In addition, assessing only a learner’s disposition for performance would not give a holistic view of the aspects desirable as learning outcomes of higher education, particularly academic teacher education.

Furthermore, Haney (2000) emphasises that the test format also influences what can be assessed, which has important consequences: In medical education, research notes that the presence of simulation-based skills assessments leads to students attending classes that train for those skills (Buss et al. 2012). In this case, the assessment format indirectly influences the learning strategies of future professionals (Gulikers et al. 2008).

Recently, some research projects started transferring their assessment methods into a digital format, often incorporating assessment feedback (e.g., Bartels and Kulgemeyer 2018; Kecker et al. 2022). However, it is unclear how this potentially changes the assessment instruments and process. Here, digital assessment might affect what areas of competence are targeted with the test instruments. In this article, we aim to gain the first insights into these questions based on examples mostly from physics teacher education. To describe the potential effects of the transfer into a digital format (including assessment feedback), we adopt an existing framework of clinical assessment by Miller (1990). Based on this, we developed a framework of teacher education assessment to describe areas of interest regarding test instruments and the corresponding assessment methods. The test instruments discussed in this paper were selected—among other reasons—because they focus on different assessment areas (e.g. knowledge and performance). For the three selected instruments, we gained deep insights into the development process of both the original and digital versions. The framework we developed serves as a heuristic to reflect on the differences and commonalities of the discussed assessment tools. Finally, we discuss the potential and limitations of the new digital methods and present our results in the form of ‘lessons learned’, which might be interesting to other researchers working on transferring their instruments into a digital format.

## Theoretical background

To develop a framework of teacher education assessment, we first identified potentially interesting assessment areas and corresponding assessment methods. Of course, we cannot give a complete overview of this extensive topic; therefore, our goal is to understand how professional competence as a goal of teacher education can be understood and how it has been assessed in recent studies. Afterwards, Miller’s (1990) framework for clinical assessment is introduced and adapted to teacher education.

### Teachers’ competence: the continuum from professional knowledge over skills to performance

Over the last decades, professional knowledge has been an essential area of research in science teacher education (e.g. Fischer et al. 2012, 2014; Ledermann and Abell 2014; Peterson et al. 1989; Van Driel et al. 1998). Here, Shulman’s (1987) work is highly influential. Over the years, three areas of professional knowledge have been identified as particularly important for teachers. It has been assumed that these three areas should be developed through teacher education and academic teacher education, in particular (e.g. Baumert et al. 2010; Cauet et al. 2015; Hill et al. 2005). First, science teachers should have sufficient content knowledge (CK), which can be understood as factual knowledge on the teaching subject. Second, their pedagogical content knowledge (PCK) includes knowledge about how to teach this teaching subject. Third, their pedagogical knowledge (PK) comprises general knowledge about teaching and learning.

Of these three, PCK has been the most researched area in science education, resulting in various frameworks such as the Refined Consensus Model (RCM) of PCK (Hume et al. 2019). Here, CK and PK are expected to be ‘foundational to teachers’ PCK in science’ (Carlson and Daehler 2019, p. 82). The RCM proposes three areas of PCK: collective PCK (which is reflected in textbooks), personal PCK (the knowledge of an individual science teacher), and enacted PCK (the knowledge and skills that solely exist in actions) (Hume et al. 2019). The RCM was developed by an international group of researchers specialised in this area to make PCK and research thereon internationally comparable.

**Fig. 1:**
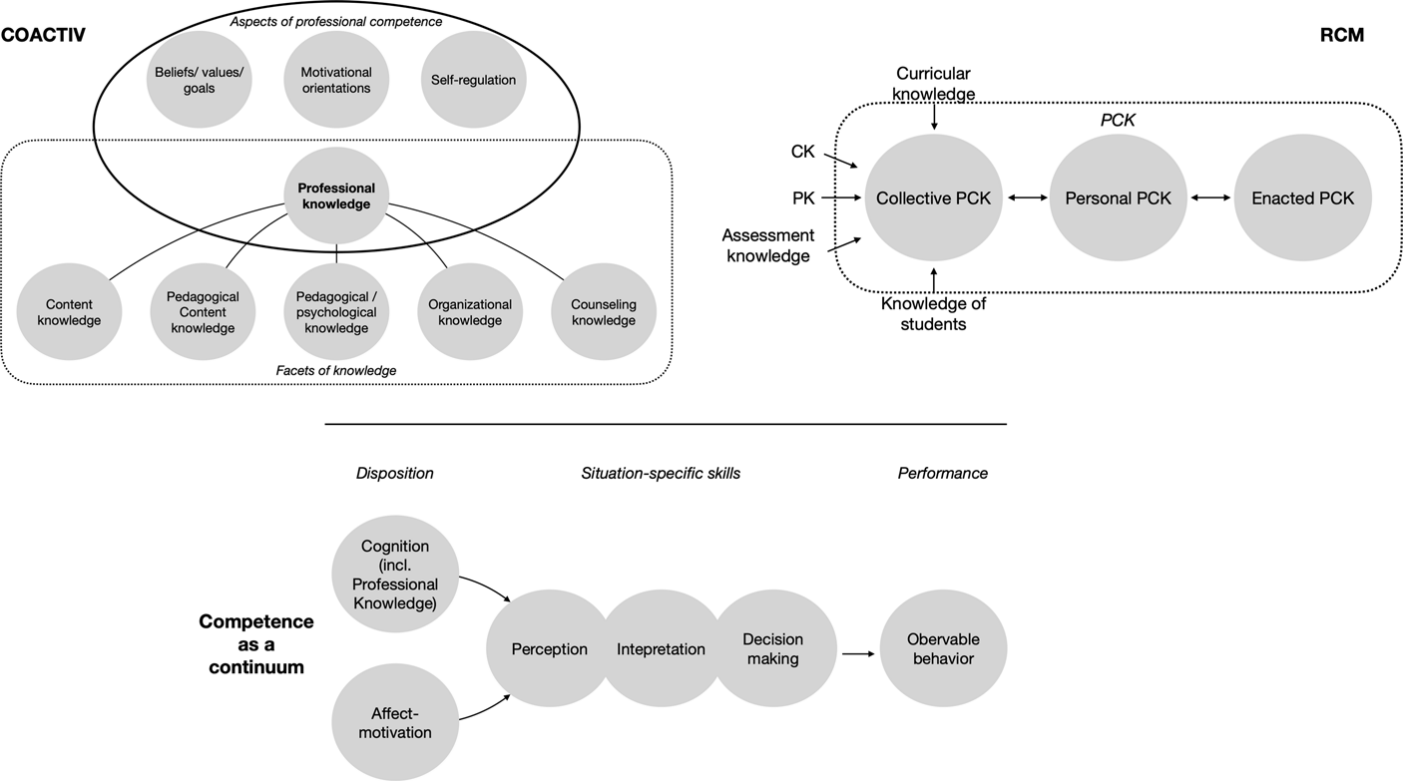
COACTIV model (*top left*; adapted from Baumert and Kunter 2013), the Refined Consensus Model of PCK (*top right*, adapted from Carlson and Daehler 2019), and the model of competence as a continuum (*bottom*; adapted from Blömeke et al. 2015). The COACTIV model addresses in more detail the disposition part of the model of competence as a continuum

Models such as the COACTIV model (Fig. 1) (Baumert and Kunter 2013) describe CK, PCK, and PK as distinct areas of equal importance in action. The COACTIV model, however, focuses on the dispositions for performance in CK, PCK, and PK. The ‘model of competence as a continuum’ (Blömeke et al. 2015) goes beyond dispositions and identifies performance as part of competence. Here, professional knowledge is the disposition for the action-related skills important in specific situations of a teacher’s profession, like in explaining or parent-teacher communication. These action-related skills ultimately result in performance. Performance should be understood as an observable outcome impacted by professional knowledge. While the part describing knowledge as a disposition is similar to the COACTIV model, the underlying idea of the part describing performance is close to the idea of the RCM, namely action-related skills are nearly identical to enacted PCK. Therefore, the three models focus on different aspects of teacher professional competence.

For this study, we use the model of competence as a continuum, because it combines essential thoughts of the RCM and COACTIV model. Here, professional knowledge represents dispositions that influence performance. However, the results will apply to the RCM with one significant restriction: we assume the existence of not just enacted PCK but also enacted PK and CK. As such, personal CK, PCK, and PK can be treated as dispositions for the enacted parts of these respective knowledge areas, rendering the RCM and the model of competence as a continuum compatible.

As a result, we believe that interesting assessment areas for teacher education should not be limited to the dispositions CK, PCK, and PK. Rather, assessment should include the more action-related parts of competence like action-related skills and performance. In addition, like in the COACTIV model (Baumert and Kunter 2013), assessment should go beyond cognitive dispositions and include other areas that might influence teachers’ actions, such as motivation or self-efficacy (e.g. Keller et al. 2017; Richardson et al. 2014).

### Assessment of different areas of teachers’ competence

Restricting an assessment to just professional *knowledge*—the disposition—would not match the broad goals of teacher education (Grossman et al. 2009). A related problem is described in physics education, namely that the relationship between professional knowledge and teaching quality is unclear (e.g. Kulgemeyer and Riese 2018; Vogelsang 2014). This situation is also found also in similar areas such as mathematics. For example, Hill et al. (2005) showed that content knowledge for teaching mathematics predicts the quality of instruction. Using the same instruments, Delaney (2012) did not find this relationship. A possible reason for this lack of evidence between disposition and performance in teaching situations is how these variables are usually measured. Most studies in this field measure teaching quality by videotaping lessons. For example, the ‘Quality of Instruction in Physics’ (QuIP) project videotaped 69 lessons of 92 participating physics teachers and analysed them for cognitive activation (Fischer et al. 2014). The ‘Professional Knowledge in Science’ (ProwiN) project (Cauet et al. 2015) analysed 23 lessons of 23 teachers for similar criteria. Therefore, both (large) research projects relied on relatively few lessons. However, the quality of a single lesson can be influenced by many variables beyond teachers’ professional knowledge (cf., Helmke 2006). Assuming that the occurrence of these variables is random, they may cancel each other out if the analysis relies on a large number of lessons. However, possibly because of the empirical effort required to analyse lessons, even large research projects such as the two mentioned above are limited to low sample sizes. Aufschnaiter and Blömeke (2010) proposed video-based testing as an alternative, but admitted that that would be closer to assessing cognition than performance. The literature provides numerous promising tools for assessment beyond knowledge tests, both for the more affective parts of disposition and action-related areas. Much research over the last decades has also focused on simulations as an assessment tool in medical education. Later, teacher education began adopting some of this research to develop performance assessments or standardised simulations (Dotger et al. 2010).

In summary, the lack of evidence between disposition and performance in teaching situations could be attributed to a measurement problem, among others. This measurement problem for assessments beyond knowledge tests has been addressed in medical education for several decades. For the area of medicine, Miller (1990) provided a framework for clinical assessment that is transferable to teacher education. Teacher education and the education of physicians share many similarities: both are academically established disciplines to prepare individuals for a particular profession and enable these future professionals to solve professional problems using evidence provided by empirical research. At least academically, both disciplines focus on developing knowledge as a disposition for more practical skills that enable a professional to deal with a broad range of professional problems (Chernikova et al. 2020). Both also provide opportunities to achieve these practical skills through, for example, field experiences and practicums.

To demonstrate the range of potential areas of assessment, Miller (1990) differentiates between the assessment of (1) knowledge, (2) competence, (3) performance, and (4) action. For Miller, knowledge first means declarative knowledge and is assessed via written tests. Assessing for competence means solving problems (e.g. written vignettes) in a primarily written form. Miller’s (1990) meaning for the term ‘competence’ is similar to that of ‘skills’ in the model of competence as a continuum by Blömeke et al. (2015) and should not be confused with the broad idea of competence in that model.

The assessment of performance focuses on acting in a more standardised environment than the assessment of action. In teacher education, this is the simulation of teaching situations. In medicine, ‘standardised patients’ acting in a specific role (Cook and Triola 2009) play a key role. There are many examples of this in medicine (Cleland et al. 2009) and related areas such as clinical psychology (Eckel et al. 2014). In general, simulations and performance assessments are an essential part of medical education (St. Pierre and Breuer 2018). We believe that if performance tests work in a complex discipline like medicine, then simulating problems should also work in teacher education (Dotger et al. 2010; Kulgemeyer and Riese 2018). In fact, science education offers various examples of these kinds of simulations. For example, Rehfeld et al. (2020) provide a systematic review of the research on teaching-learning-laboratory seminars. These seminars usually consist of two phases: (1) groups of pre-service teachers developing instructional material theoretically, and (2) testing the instructional material with small groups of high school students in settings of reduced complexity. Fischer et al. (2021) use a simulated biology classroom to diagnose procedural knowledge and research and its connection to declarative professional knowledge. Furthermore, De Coninck et al. (2019) describe the development of two simulation-based learning environments for parent-teacher communication (an online simulation and a face-to-face simulation). Mikeska and Howell (2020) describe how pre-service teachers engaged student avatars in a simulated classroom in scientific argumentation. Finally, Nilsson and Karlsson (2019) found that content representations (CoRe), together with video annotation, helped pre-service teachers develop a better quality of reflection-on-action. This is an example of simulating a guided (scaffolded) reflection process.

In medicine, a set of such simulations is often referred to as an ‘objective structured clinical examination’ (e.g. Harden et al. 1975), for example, including pelvic examinations (Rochelson et al. 1985) and psychiatry (McNaughton et al. 2008). However, reliable test scores are not easily derived from these assessment formats. The usual way to achieve test scores involves observers and scoring sheets, an approach demonstrated as having sufficient reliability, objectivity, and validity (Hodges et al. 1998; Walters et al. 2005). There are also examples of such simulations in teacher education, such as in explaining situations (Bartels and Kulgemeyer 2018; Kulgemeyer and Riese 2018; Kulgemeyer and Schecker 2013).

The last and most complex area of Miller’s (1990) model is the assessment for action or action quality, which involves acting in a professional situation with all its complexity. In teacher education, there are numerous examples of this type of assessment using real observed lessons (e.g. the abovementioned projects QuIP [Fischer et al. 2014] and ProwiN [Cauet et al. 2015], and COACTIV [Baumert et al. 2010]). We believe that the different areas of assessment in Miller’s (1990) model—assessment of knowledge, competence, and performance—have implications for teacher education. The academic part of teacher education aims to develop teachers’ knowledge and competence to enable them to achieve high-quality action in their first teaching experiences in their pre-service period (KMK 2009). For a holistic view on teacher education and *supervision* of teacher education, all parts of teachers’ competence should be assessed, which fits well with Miller’s (1990) understanding of assessment areas.

Furthermore, Miller’s (1990) framework adds a vital perspective to the models of professional competence discussed earlier. Ultimately, Miller (1990) applied a straightforward approach to characterise different assessment forms; however, he did not link them to a model of professional competence. Models of professional competence describe the latent variables that can be targeted in teacher education. Miller (1990) describes how similar the problems to be dealt with in a particular assessment format (the manifest variables) are to problems in the real-world professional context. However, he distinguishes between assessments of performance and of action, with the first including acting in a standardised environment. In the model of competence as a continuum, both assessment formats target the latent variable of performance, and in the RCM, both target the latent variable of ‘enacted PCK’. Kulgemeyer and Riese (2018) showed that testing for performance enables controlling many factors confounding the effects of professional knowledge on the instructional quality. This may have been a problem in ProwiN and QUIP. Therefore, distinguishing between performance and action seems plausible for educational research and teacher education: learning how to act under controlled conditions and in settings with reduced complexity might be the first step to being able to act in all types of professional situations. Therefore, we believe that like in Miller’s (1990) model of clinical assessment, a framework of teacher education assessment makes sense because it adds the two distinct parts of performance and action to the models of professional competence. However, we acknowledge that this leads to a focus on assessment formats and therefore, on manifest variables. Thus, it remains unclear whether to distinguish between performance and action as latent variables.

### A framework of teacher education assessment

We use Miller’s (1990) framework of clinical assessment to develop a framework of teacher education assessment. The goal of our framework is to provide a sufficient basis for discussing and situating different assessment methods, and to discuss their potential place in teacher education. We also aim to demonstrate heterogeneous assessment methods. While this article focuses on digital assessment methods, the framework should be helpful in differentiating between assessment methods in general.

The teacher education assessment framework consists of two dimensions, which we call ‘assessment areas’ and ‘assessment methods’. The assessment areas mirror what Miller (1990) proposes for clinical assessment. It is assumed that these assessment areas represent the important constructs teacher education aims to develop. Therefore, all these constructs have different learning opportunities in the teacher education system, and are located in the different phases of the (German) teacher education system. In this article, we focus on the academic part of education as provided at universities. However, we acknowledge that the idea of life-long learning comprises further development of these constructs that correspond with the assessment areas also for in-service teachers and the second part of (German) teacher education, namely the induction phase (‘Referendariat’).

The second dimension comprises assessment methods. This is by no means a complete list of all possible assessment methods. Here, the dimension is limited to the probably most frequent assessment formats used in physics teacher education. Depending on their primary goal, every assessment method can be classified under one or more categories of this dimension. Most important, every assessment method focuses on one or more assessment areas and therefore, on a different part of professional competence. In the following, we elaborate on these dimensions.

#### Dimension ‘assessment areas’

For our framework of academic teacher education assessment, we accord with Miller’s (1990) differentiation of the areas that can be assessed. However, to differentiate Miller’s (1990) terminology from that in the model of competence as a continuum, we use the term ‘skills’ for Miller’s (1990) ‘competence’. We believe that Miller’s (1990) use of ‘competence’ is close to the skills in the model of competence as a continuum and that this model has a broader idea of what competence comprises.

In general, the assessment areas Miller (1990) describes—knowledge, competence, performance, and action—have a different degree of complexity and authenticity in terms of being similar to everyday classroom experiences. This idea is expressed by authors including both Terhart (2012), Diez (2010), and in Blömeke et al.’s (2015) model of competence as a continuum. Here, knowledge can be assumed as a disposition for the skills that are the basis for performance and action quality in various situations of a teacher’s (professional) working life.

As mentioned, we acknowledge that the different parts of (German) teacher education play different roles in acquiring the constructs reflected in the ‘assessment areas’ dimension. While the academic part of teacher education focuses on knowledge and skills in their most frequent teaching forms, lectures, and seminars, the induction phase has a more practical focus and aims to develop action quality. Still, academic teacher education provides opportunities for field experiences and in some cases, for simulated teaching or microteaching in teaching-learning laboratories, for example. Both forms of learning opportunities have been increasingly used over the last years (Kaufman and Ireland 2016; Krüger et al. 2018; Rehfeldt et al. 2020). Essentially, different learning opportunities exist for the constructs that teacher education aims to develop.

To determine the assessment targeted by an assessment instrument, the levels of both complexity and authenticity are essential criteria (Table 1).

**Table 1:** Criteria for assessment areas

Assessment Area	Level of authenticity/complexity	Examples
Declarative Professional knowledge/Attitudes	Low level of authenticity and low level of complexity	Naming important misconceptions for Newton’s third law
Skills	Low level of authenticity, middle level of complexity	Diagnosing misconceptions in authentic teaching vignettes (and proposing how to proceed in teaching)
Performance	High level of authenticity, middle level of complexity	Dealing with a limited number of professional problems under standardized conditions (e.g., explain a physics topic to a simulated student)
Action	High levels of authenticity and complexity	Dealing with various professional problems in a professional context (e.g., teach a class in physics)

For example, action differs from performance in terms of the level of complexity. While performance assessments use standardised conditions, ‘action’ means acting in a professional environment (e.g. teaching a class in physics), which usually involves dealing with many professional problems at the same time (e.g. diagnosing misconceptions, establishing a sufficient learning environment, and moderating a discussion). ‘Performance’ and ‘skills’ differ in their level of authenticity. While both may target one or two professional problems at the same time, ‘performance’ would involve real action (e.g. face-to-face explanations). ‘Skill’ comprises hypothetical action (‘if I were in the same situation as the teacher in the video vignette, I would do the following …’). This usually requires a lower level of ‘action under pressure’, which is typical for a high level of authenticity in teaching.

#### Dimension ‘assessment methods’

Concerning typical assessment methods in teacher education, to illustrate our framework, we differentiate between written tests, tests involving video vignettes, performance assessments involving simulations and demonstration lessons, and videotaped lessons. These methods are examples, which, while focusing on different assessment areas, do not exclude other assessment areas. For example, the assessment area depends on the types of items for written tests. Situational judgement items measure the area of skills, while some multiple-choice items are limited to the area of knowledge in Miller’s (1990) sense. We believe that these assessment methods at least represent the typical methods used in physics teacher education for formative and summative assessment. To our knowledge, no studies provide an overview of the extent to which different assessment methods are used in physics teacher education.

**Fig. 2:**
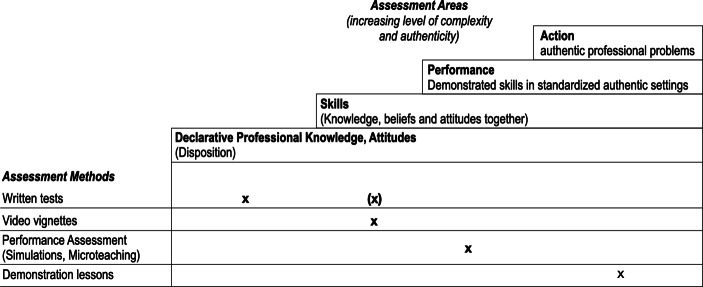
Model of teacher education assessment in academic teacher education. The bold x represents the parts of the model we analyse in Sect. 3 (e.g.: knowledge in the written CK test, skills in the written PCK test)

Figure 2 shows the framework for teacher education assessment. It accords with Miller’s (1990) framework and adds possible assessment methods as a dimension to the parts of professional competence to be assessed. Again, we highlight that this framework is open for further development and certainly does not cover all possible approaches (e.g. oral exams, essays, field reports). However, we believe that it serves as a heuristic to describe different assessment methods and the areas they aim to test. In particular, it shows how broad potential (digital) assessment methods could be.

## Developing digital assessment methods for physics teachers’ professional competence

In this section, we first describe the effects of transferring assessment instruments for a specific part of physics teachers’ competence into a digital format using three examples. The three assessment tools were selected because they cover different parts of the framework in Fig. 2. While the framework for explaining performance is located in the assessment area of performance, the test for PCK is located in both professional knowledge and skills, and the test for CK is mostly located in the assessment area of professional knowledge. Therefore, by choosing these examples, we cover a broad range of assessment areas (Fig. 2). Furthermore, the three examples were selected because we gained deep insights into the development of the test instruments both in the original and the digital format, and, thus, we can share our lessons learned. Second, we added an additional perspective from other subjects by including two more examples in the exploration: the test for German as a foreign language and a simulation of parent-teacher communication. These two examples were chosen because like the test for explaining performance, they target aspects of communication and, in this respect, are comparable. As such, they add perspectives from other subjects. Therefore, the sample for the exploration consists of three instruments used for various aspects of communication: one covers the assessment areas from skills to performance and two the dispositions from knowledge to skills.

### Assessing physics teachers’ explaining performance (original assessment area: performance)

Explaining situations can be understood as a core part of instruction (Geelan 2012). However, it should not be confused with a non-constructivist teaching approach (Kulgemeyer 2013; Kulgemeyer 2019). Providing instructional explanations requires adaptation to the prior knowledge and therefore, interaction between explainer and explainee(s) to diagnose the state of knowledge and success of explanation attempts (Wittwer and Renkl 2008). Furthermore, instructional explanations have their place in science classrooms, particularly with learners with low prior knowledge, and should be embedded in ongoing mental activities allowing an application of the explained principles in well-designed learning tasks (Kulgemeyer 2019). Explanation attempts containing a simple presentation of knowledge most likely fail (Wittwer and Renkl 2008). Evidence shows that teachers who consider instructional explanations transmissive and not a constructivist practice tend to give less successful explanations (Kulgemeyer and Riese 2018). Therefore, providing effective instructional explanations is not only a core part of science teaching, but also a challenging practice (McDonald et al. 2013).

We developed a test instrument to assess explaining performance (Kulgemeyer and Schecker 2013). It consists of a simulation of a dialogic explaining situation in which a student asks a teacher for further clarification on a physics topic. The participants play the role of the teacher in a standardised setting with a given time and pre-designed material (e.g. diagrams, etc.). The most essential standardisation is revealed after the test: the students, who play the role of explainees, have been trained to behave similarly and ask standardised questions or give standardised prompts to adapt the explanatory attempts to the knowledge state of the individuals. They are ‘standardised students’ following the example of standardised patients in the abovementioned performance assessments in medical education (Dotger et al. 2008). Multiple studies provide evidence for good validity and reliability (Kulgemeyer and Tomszyczyn 2015). However, the data analysis here requires much time and effort, as the videos of these situations must be analysed using categories matching a model of explaining science (Kulgemeyer and Schecker 2009). To enable large-scale assessments, a digital version of this instrument has been developed (Bartels and Kulgemeyer 2018; Bartels et al. 2019).

**Fig. 3:**
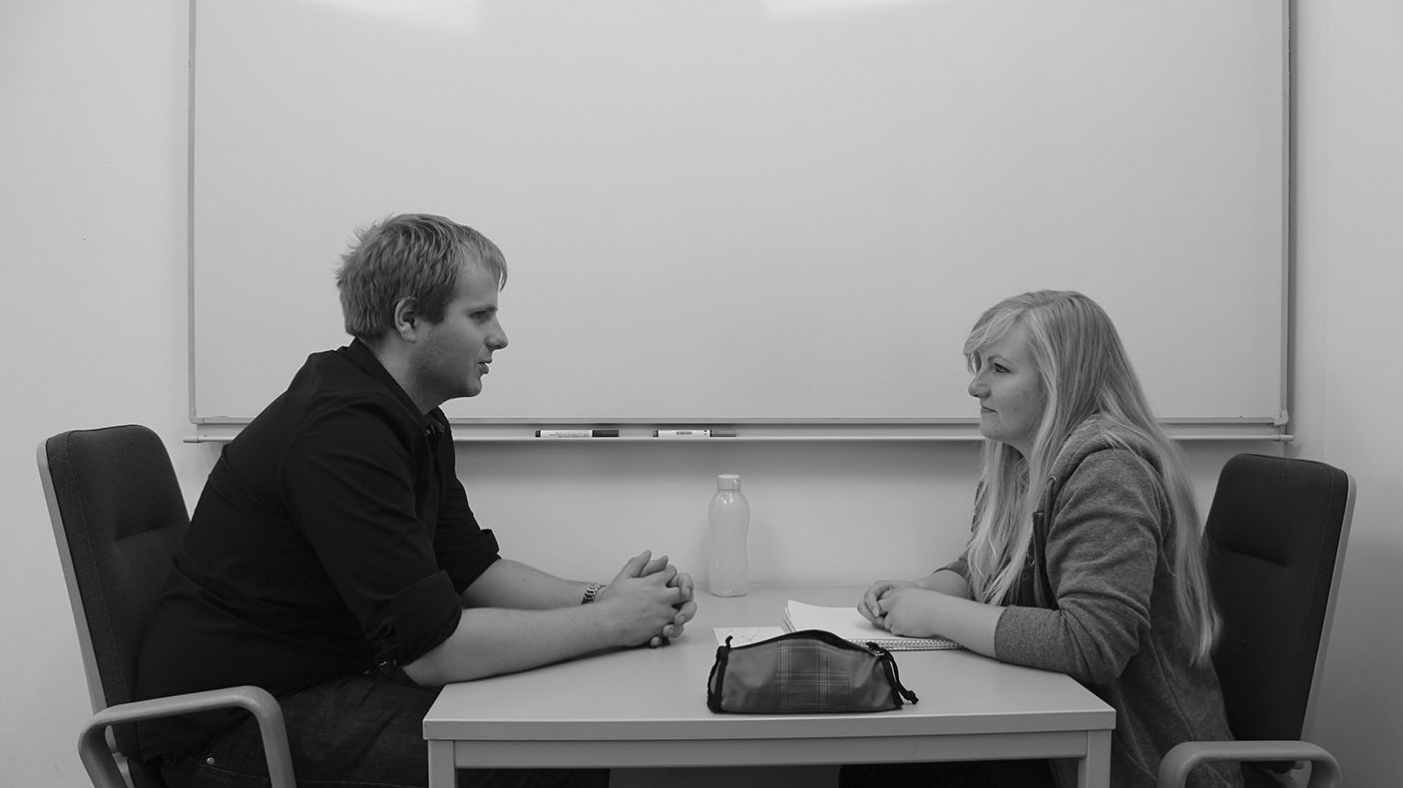
Screenshot of a video vignette

For this purpose, the goal was to develop multiple-choice items. We filmed a staged explanation dialogue between a teacher and a student, and the participants had to decide after each turn of the dialogue how the teacher should proceed. To develop distractors and attractors for this instrument, the qualitative data of the original instrument was analysed (explaining dialogues from *N* = 198 participants), and statements that appeared during these dialogues were used. Therefore, all the attractors and distractors appeared in one of the explaining dialogues before, and are, therefore, authentic. Changes had to be made to the actual wording to make the attractors and distractors more comparable. We considered this step in developing authentic answers in closed item formats as crucial in retaining the focus on performance. A think-aloud study suggested that this goal was achieved: the participants perceived the dialogues as authentic and felt their desired answers were included in the distractors and attractors. The final instrument consists of two-tier items, which have previously been described as promising in measuring beyond knowledge (e.g. Tan et al. 2002) (Fig. 3). After deciding how to proceed (Tier 1, see below), the second tier included reasoning this decision (Tier 2, see below). Different studies confirm the high validity and sufficient reliability of this approach (Bartels et al. 2019). The final instrument is accessible online in German and English (explainingscience.de). It has been used in physics teacher education in Germany and Australia. The latest version also provides assessment feedback for participants regarding their performance compared to the baseline of all participants (Bartels et al. 2019) and advice for the further development of their skills.

Next, we present a shortened sample item (taken from Bartels et al. 2019) taken from a dialogue between the teacher Mr Miller and student Sarah right after Sarah signalled that she did not understand the principle of superposition. Mr Miller now askes her to elaborate:

Mr Miller: ‘Can you tell me what part you did not understand?’—Sarah: ‘I’m not sure; there were so many science terms … could you explain the principle of superposition in an easier way so that I can understand it?’

Tier 1 (single choice, shortened)

1. The principle of superposition describes how two forces that apply at different angles must be summed up. The result is another force that points in the direction of the body’s actual acceleration. This is exactly what happens to the parts of the asteroid.

2. Let’s have a closer look at this. First, the asteroid moves straight toward the earth and has a certain velocity. When it blows up, the asteroid is separated into two parts that diagonally pass the earth on the right and left. Therefore, the earth is missed, and mankind survives.

3. We just found out that each part of the asteroid has two parts of its velocity pointing in different directions. As the asteroid moves towards the earth, one part points towards it. The second part is added by the detonation and points sideward. Now, the principle of superposition says that these two velocities are added to give the object’s velocity. Therefore, the parts of the asteroid move forward diagonally.

Tier 2 (multiple-choice, shortened)

1. Use language that fits the student’s language level.

2. Use language with appropriate physics terms.

3. Use a context that might be interesting for students

Validity studies suggest that the original and digital versions are not interchangeable. Based on an analysis of a nomological network, Bartels et al. (2019) conclude that the digital version provides good insights into a core practice of physics teachers, but the simulation of the dialogue is more authentic and includes a broader range of skills. For example, they report correlations to epistemological beliefs and PCK comparable to those found in the original version. Still, they argue that the degree of freedom in a real-world professional situation is reduced. The limitations of the digital instrument are in the simulation of real interaction. The focus shifts from being the protagonist to being an observer, even though decisions for the teacher in the video test need to be made. For example, this is expressed by the less than perfect correlation between the original and digital test format (*r* = 0.47, *p* < 0.05).

### Assessing physics teachers’ pedagogical content knowledge (original assessment area: skills)

The written test to measure physics teachers’ pedagogical content knowledge has been used in various studies (e.g. Gramzow 2014; Kulgemeyer and Riese 2018). The underlying model for pre-service physics teachers’ PCK considers different influential conceptualisations of PCK in the Anglo-American tradition (e.g. Lee and Luft 2008; Magnusson et al. 1999; Park and Oliver 2008) and German tradition (e.g. Baumert et al. 2010; Riese and Reinhold 2012). Also relying on curricula analyses, a structural focus centred on the PCK students could acquire in academic teacher training programs in Germany. The focus regarding the physics content area was on mechanics. The final model comprises four subscales of PCK: (i) instructional strategies, (ii) students’ misconceptions, (iii) experiments and teaching of an adequate understanding of science, and (iv) PCK-related theoretical concepts. To create items with different requirements and difficulties, the model also covers the following cognitive activities: (i) reproduce, (ii) apply, and (iii) analyse.

**Fig. 4:**
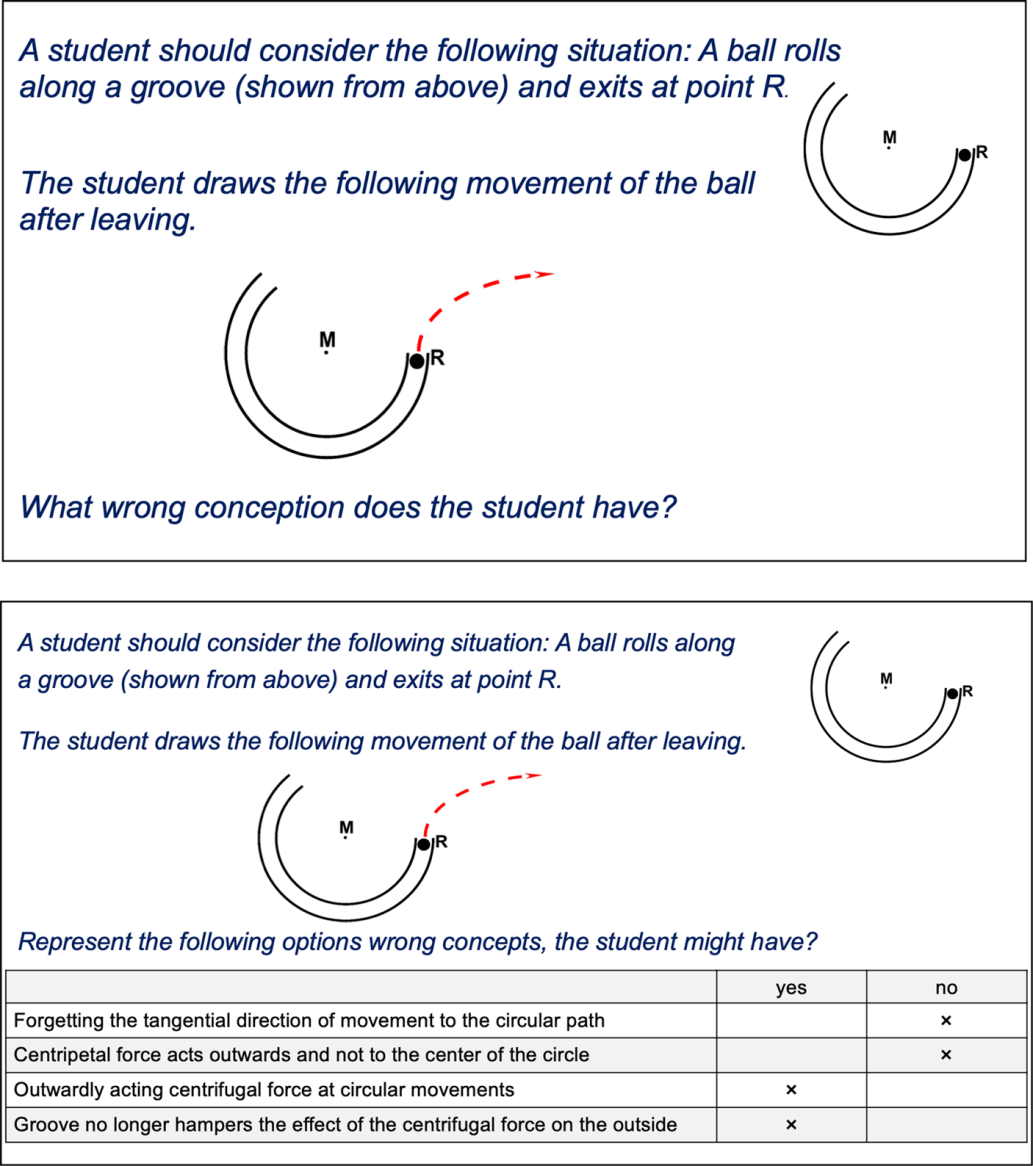
Comparing items from the original version (*top*) and digital version (*bottom*)

Based on this model, a paper-and-pencil test including 43 items (60 min) with open situational judgement items and complex multiple-choice items (multiple select) was developed. Students’ responses to the open items of the test were rated manually using a comprehensive coding manual. This results in much time and effort spent on analysis, which is acceptable for research purposes but considerably complicates the transfer in academic teacher education for education purposes. Therefore, the test was further developed into an online-based test comprising 31 complex multiple-choice items (multiple select). While both the introductory texts and instructions could be adapted from the paper-and-pencil test instrument (except for a few adjustments regarding the wording), many distractors and attractors had to be developed (143 options to answer altogether). Therefore, for each item, the available answers to the open-ended items from 52 students were used to develop attractors and distractors. The digital version has been implemented into the freeware online assessment environment LimeSurvey (Fig. 4).

We found ambiguous evidence regarding the comparability of the original and digital versions. For the four subscales of the test, the correlations between the test formats range between *r* = 0.37 (*p* < 0.05, subscale *experiments and teaching of an adequate understanding of science*) and *r* = 0.81 (*p* < 0.01, subscale *PCK-related theoretical concepts*) in a sample of 24 physics students. We concluded that some items of the original test (in particular open situational judgement items) require a broader range of skills: for example, some open judgement items require describing possible reactions to students’ statements. The participants only chose their reactions from different options given in the digital version. We believe that the original version with open situational judgement items collects valid data when a broad coverage of the underlying construct is required. The digital PCK test has its strengths when instant feedback is required, such as for teaching purposes.

### Assessing physics teachers’ content knowledge (original assessment area: knowledge)

We designed the original paper-and-pencil test to measure content knowledge in mechanics. The instrument consists of 48 single select items (Enkrott 2020). The test-taking time was 50 min.

The original research purpose of the instrument was to enable a differentiated measurement of learning gains from the beginning of the bachelor’s program to the end of the master’s program in the three dimensions of physics content knowledge: (1) school knowledge (SK), (2) university knowledge (UK), and (3) deeper school knowledge (DSK). The underlying model differentiates SK and UK by referring to the curricular order of the physics content (e.g. the oblique throw = school physics; flux = university physics) and advanced level of mathematics (e.g. differential equations = advanced [UK], linear equations = basic [SK]). DSK is defined by specific content-related processes that should be useful for teaching (e.g. ‘identify derivation and solution approaches’, ‘dealing with model limitations’).

As a CK test, the instrument measures cognitive dispositions (cf. Fig. 2). We assumed that university students acquire most of this knowledge explicitly (SK, UK) or implicitly (DSK) via physics lectures and seminars. However, Enkrott (2020) showed that students also improved on this scale through practical teaching experiences.

The purpose of the original paper-and-pencil test was to collect data for research. However, the new online CK assessment primarily gives feedback to teachers and students. The new digital assessment accounts for this new purpose. We condensed the item basis to 22 items in the final online test, reducing testing time by more than 50%. Furthermore, the new choice of items allowed us to construct a one-dimensional mechanics knowledge assessment from this instrument with two difficulty levels (basic level = SK; advanced level = DSK and UK).

The new test is so similar that we considered it reasonable to perform the validation on the original paper-and-pencil data. For the item selection (*N* = 482), we calculated a weighted likelihood estimate (WLE) reliability of 0.80. The EAP/PV reliability of 0.82 is comparable to the original version. Based on the item difficulty, we also reproduced the two levels: ‘basic’ and ‘advanced’. Instead of choosing an arbitrary cut-off value, we labelled the overlapping region as intermediate.

**Fig. 5:**
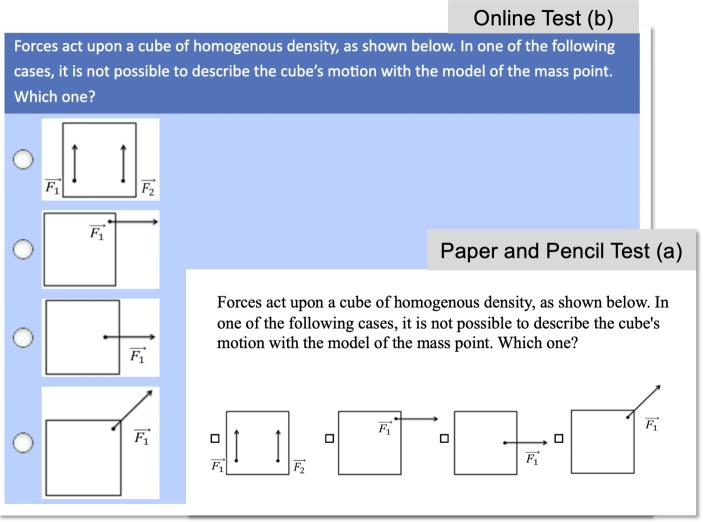
Comparing items from the original (*right*) and digital (*left*) version

As the original test is a multiple-choice instrument, developing the digital version was straightforward. It did not impact the nature of the test-taking situation (cf. Fig. 5).

### Assessing skills for German as a foreign language (original assessment area: skills)

The ‘DaF-test’ has been used as a paper-and-pencil version since 2001 to assess applicants for a German university on their skills in German as a foreign language (Kecker et al. 2022). Since 2020, the test has been available in a digital format. Both versions cover four aspects: reading, listening comprehension, writing, and speaking. In this case, the integration of media (e.g. videos for listening comprehension) has a different effect: ‘The change of communication in the digital age can be covered more authentically and more precisely in a digital format compared to the paper-and-pencil version’ (Kecker et al. 2022; p. 394, translation by the authors). In this case, the digital format also leads to a more standardised assessment and therefore, increases the objectivity thereof. Therefore, the higher authenticity of the digital format stems from the fact that communication itself is becoming more digital.

### Simulating parent-teacher communication (original assessment area: performance)

De Coninck et al. (2019) describe the development of a simulation of parent-teacher communication in both a digital format and face-to-face-prototype. It is important to highlight that this simulation is meant as a learning environment, but is similar to the face-to-face communication test described above regarding explaining performance and therefore, provides an interesting comparison. In addition, these simulations can be used for assessment purposes with slight modifications.

The face-to-face communication prototype includes two parts: ‘(1) reading an introduction to the case and (2) engaging with standardised individuals in a real-time simulated parent-teacher conference’ (De Coninck et al. 2019, p. 271). All standardised individuals were trained for their roles. The online simulation had a different structure. After reading an online case description, the participants watched video clips of a simulated parent-teacher conference. These clips are paused at times, and participants are asked what they would do as the teacher.

De Coninck et al. (2019) conducted a study to compare both formats. While both formats were generally positively evaluated, the participants rated face-to-face communication as more authentic (De Coninck et al. 2019, p. 273).

## Rating the assessment areas of the instruments

**Fig. 6:**
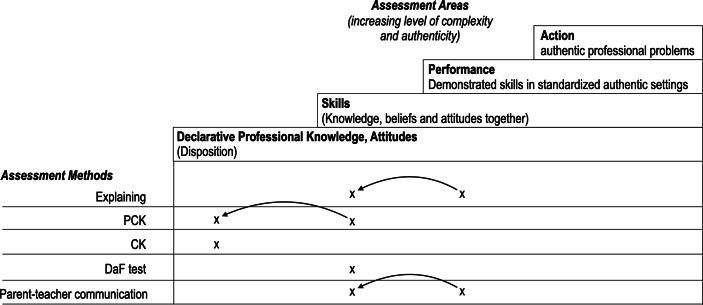
Categorising the assessment tools on the framework. The *arrows* represent the change from the original version to the digital format

The five examples provided have different foci concerning the framework of teacher education assessment. However, we believe that the switch from a traditional form of assessment to a digital approach changes these foci (Fig. 6).

The traditional test for explaining performance, the dialogic explaining assessment, focuses on performance as an area of assessment. It provides enough space for participants to act authentically under standardised conditions (Dotger et al. 2010), which implies a high level of authenticity (cf. criteria in Table 1). However, it has a much narrower focus on one particular teaching situation, and does not assess teachers’ instructional quality in a broad sense. Regarding the criteria in Table 1, this narrower focus leads to a middle level of complexity rather than the high level required by the assessment area ‘action’. The switch to the digital format has consequences. To give instant assessment feedback, the item formats were changed to two-tier multiple-choice items. Even though decisions for the teacher in the videos had to be made in these items, this is a much less authentic situation than acting as a teacher in the original version. Furthermore, the participants act more as observers than as explainers. Referring to the criteria in Table 1, this results in a shift in the assessment areas of the framework of teacher education assessment from performance to skills. Bartels et al. (2019) introduce the term ‘performance-oriented assessment’. They argue that the assessment is still closer to action than a written competence test, as the instrument bridges the gap between skills and performance. The shift to digital assessment resulted in a less authentic format for the benefit of more automatic scoring and therefore, the potential to use the instrument in large-scale assessment. Possibly, an analysis of verbal data from a direct dialogue with a digital avatar using natural language processing and machine learning might prevent this shift in the future (Zhai 2021; Zhai et al. 2020). Regardless, this would still be a less authentic situation as long as the appearance of the avatar can be distinguished from humans.

We reached similar conclusions regarding the PCK test. The original version of the test includes multiple-choice and situational judgment items. The latter, however, include written vignettes of complex teaching-related problems that require written answers on how to act as a teacher in the given situation. It can be argued that this is more than a ‘declarative professional knowledge’ test in the ‘assessment areas’ dimension of our model. Regarding the criteria in Table 1, these situational judgment items have a higher level of complexity than the declarative professional knowledge items. However, they provide a lower level of authenticity than the assessment area ‘performance’ would require, because participants must decide how a fictional teacher should act instead of acting under pressure themselves. The shift to a digital instrument had a similar effect on the explaining performance test: the open item formats were reduced, and more multiple-choice items were introduced, which enabled an automatic analysis of the tests. However, again, the level of authenticity was decreased. While in the open items, participants had no help in their decisions, the closed items proposed different solutions of which they had to select one (the attractor of the item) (cf. Fig. 4). Thus, it can be argued that the digital version is primarily located in the assessment area of ‘knowledge’, while the original version is in the assessment area of ‘skills’. Furthermore, future use of machine learning might prevent this shift regarding this instrument, as it allows analysing the written test and automatic scoring.

This effect was not observed for the CK test. The original version included multiple-choice items and is located in the assessment area ‘knowledge’ among the criteria in Table 1. It did not require transformations that rendered the test-taking situation less authentic or complex.

For the DaF-test, the digital version has increased authenticity. The reason, however, is that communication itself has become more digital, an aspect the test needs to cover. Regarding the criteria in Table 1, both the original paper-and-pencil version and digital format of the test have a moderate level of complexity. The four aspects of reading, listening comprehension, writing, and speaking are tested separately, although ‘real’ communication would likely include a mixture of two or more of these aspects. The level of authenticity ranges between low and moderate in terms of the criteria in Table 1. While some items require open answers (e.g. talking to another person), these answers are minor parts of a conversation. Therefore, both the original and digital versions can be classified under the assessment area of ‘skills’ although they are close to ‘performance’ as well.

Regarding the parent-teacher communication simulation, the case is similar to the abovementioned explaining performance assessment. The original version involves acting under standardised conditions with simulated parents, while the digital version includes a shift of perspective: participants must now watch a video and decide for the teacher in the video what they would do in his/her case. Therefore, the original version has a high level of authenticity and moderate level of complexity (assessment area performance). The digital version has a low level of authenticity and retains a moderate level of complexity (assessment area skills).

## Potenzial and limitations: what can we learn from these examples?

For these examples, it seems that the transfer to a digital format mostly leads to less authenticity, except for knowledge tests. However, the DaF-test example shows this is not automatically the case.

The concept of authenticity and its role for validity is underdeveloped, but important when dealing with assessment formats in teacher education (Dotger et al. 2008). Less authenticity is a potential validity problem (Stadler et al. 2021). It remains an open question how authentic a situation needs to *seem* (not necessarily: needs to *be*)—in terms of the model of competence as a continuum—to activate action-related skills to deal with problems. However, what we know from situated learning indicates that authentic ‘anchors’ are important parts of an educational approach that results in knowledge that can be applied in professional contexts (e.g. Brown et al. 1989). This suggests that an assessment that focuses on various aspects of professional competence should have an authentic anchor to stimulate skills that are important for professional teaching. We advise proposing arguments for authenticity in every assessment method ‘beyond knowledge’ as part of their validity. This may be an essential aspect of content validity. Furthermore, the increasing number of assessment formats may mean that different areas of teachers’ competence are less reflected in education.

On the other hand, these digital assessments enable direct assessment feedback and, therefore, may provide the chance to better use assessments as learning opportunities for teacher education. The development of assessment formats beyond knowledge seems critical for teacher education in general for various reasons including better supervision of the results of a teacher education system. Essentially, our lessons learned are as follows:

1. Assessment as part of education should focus on different aspects of teachers’ competence. These aspects are included in the model of teacher education assessment. Furthermore, digital assessment can (potentially) cover all aspects included in the framework.
2. Developing digital tools with multiple-choice items from open-ended items helps provide instant assessment feedback. This is an often-mentioned advantage of digital assessment (e.g. Casanova et al. 2021). Both the explaining performance instrument and PCK instrument use large datasets of answers to open-ended items to develop authentic distractors and attractors.

Of course, general test quality criteria apply to both the digital format and more traditional ways of assessment. Neumann et al. (2019) recognise the potential of digital assessment as it allows new approaches: ‘These include touch screens with drag and drop and multi-touch features, augmented reality (AR), virtual reality (VR), mixed reality (MR), robots, and behavioral monitoring (e.g. voice recognition, eye gaze, face recognition, touchless user interface)’ (Neumann et al. 2019, p. 1). In an early literature review, Wall (2000) points out several advantages of the digital format, including higher accessibility, the potential to give immediate feedback, more efficiency in testing, helping persons with disabilities, and assessing higher-order skills. The latter includes performance and action in our framework. Wall (2000) notes, ‘Test developers can construct situations that simulate the real world’. However, Wall (2000) compares digital assessment only with paper-and-pencil tests, and does not mention performance assessments. As one core disadvantage of digital assessment, Wall (2000) highlights that it lacks human contact. As such, a high awareness of the advantages and disadvantages is needed to successfully use digital assessment. Wall (2000) mentions numerous disadvantages regarding data security. We believe that test developers should also be critical regarding the use of digital simulations of human interaction. A question for future research is what the limits of authenticity are. This leads to our third lesson learned:

3. We highlight that we could only analyse five examples, and they might not be representative. The selection of the cases, therefore, contributes to this last lesson learned. The analysis reveals, for these cases, that digital tools tend to be less authentic than analogue tools when based on simulations. This might affect which aspects of professional competence can be targeted with these tools, as the aspects closer to action require authenticity as a core part of validity. These results highlight the potential of technologies such as VR, machine learning (AI), and natural language processing to mitigate this effect (e.g. by automatically scoring text items). However, we are still far from mimicking the complex nature of human interactions, a core element of the teaching profession.

To sum up, we believe more digital assessment tools should employ the advantages of instant assessment feedback. This would be helpful for teacher education if the assessment focuses on a broad range of areas including those ‘beyond knowledge’. However, the measure of validity should include a measure of the authenticity of the assessment method. In particular, digital tools might be less authentic in terms of human interaction, and as such, a challenge for future research is to determine what the limits of authenticity are.
